# Polycomb Group Protein Bmi1 Is Required for Growth of RAF Driven Non-Small-Cell Lung Cancer

**DOI:** 10.1371/journal.pone.0004230

**Published:** 2009-01-19

**Authors:** Matthias Becker, Christian Korn, Arnold R. Sienerth, Robert Voswinckel, Katharina Luetkenhaus, Fatih Ceteci, Ulf R. Rapp

**Affiliations:** 1 Bayerisches Krebsforschungszentrum (MSZ), University of Wuerzburg, Wuerzburg, Germany; 2 University of Giessen Lung Center, Department of Internal Medicine, University Hospital Giessen, Giessen, Germany; Emory University, United States of America

## Abstract

**Background:**

We have previously described a RAF oncogene driven transgenic mouse model for non small cell lung cancer (NSCLC). Here we examine whether tumor initiation and growth requires the stem cell self-renewal factor Bmi1.

**Principal Findings:**

In order to evaluate Bmi1 function in NSCLC two founder lines that differ in incidence and latency of tumor formation were compared. Ablation of Bmi1 expression in both lines had a dramatically decreased tumor growth. As the line with shorter latency matched the life span of Bmi1 knock out mice, these mice were chosen for further study. The absence of Bmi1 did not decrease the number of tumor initiation in these mice as only the size and not the number of tumors decreased. Reduction in tumor growth resulted from an increase in cell death and decrease in cell cycle progression that corresponded with up-regulation of the p16^INK4a^ and p19^ARF^.

**Significance:**

The data identifies Bmi1 as an important factor for expansion but not initiation of RAF driven NSCLC.

## Introduction

Lung cancer is the leading cause of cancer death in the western world. Based on histology two different types of lung cancer, non small cell lung cancer (NSCLC) and small cell lung cancer (SCLC) are defined. Overall 50% of all adenocarcinomas, the most prevalent form of NSCLC, harbor mutations in constituents of the mitogenic signaling cascade i.e EGFR, K-Ras and B-RAF [1]. Mutations of C-RAF are also found in NSCLC, however, at a lower frequency.

Several systems that model lung cancer in the mouse have been reported. The strategies to establish these models have basically followed two different paths. 1) Induction of oncogene expression and deletion of tumor suppressor genes via adenovirus directed expression of Cre-recombinase [2]–[5]. This approach mimics occurrence of somatic mutations in adult tissues. 2) Constitutive oncogene expression in target cells through the use of cell type specific promoters [6], [7]. The latter models may be more appropriate for gestationally acquired mutations. While the virally induced models show a somewhat undefined target cell spectrum the transgenic approach has the potential of targeting all the transformation sensitive cells that show expression from a particular cell type specific promoter.

We previously described the generation of transgenic lines expressing an oncogenically activated version of the C-RAF kinase (C-RAF BXB) under the control of the human alveolar type two (AT2) cell specific surfactant protein C (SP-C) promoter [7]. Two founder lines, further on referred to as BXB11 and BXB23, show lung targeted adenoma development with high penetrance, high incidence and short latency. Tumors are phenotypically rather stable as no nuclear atypia or metastasis was seen on a C57Bl/6 background. A cuboidal cell type predominantly forms the adenomas. No bronchial epithelial hyperplasia continuous with cuboidal tumors as described for oncogenic K-Ras models [4] has been observed. The BXB23 founder line has been used extensively in genetic studies to analyze tumor progression influencing factors [7]–[12]. Although the transgene expression in principal targets all AT2 cells only a subset of them responded to expression of the C-RAF BXB with the formation of tumors [7]. One explanation of this finding is that a lung progenitor compartment responds to mitogenic cascade signaling with expansion. Alternatively shut down of transgene expression in the majority of AT2 cells may occur. We favour the first possibility and assume that this progenitor compartment is Bmi1 positive and depends on mitogenic cascade signaling for self-renewal since treatment with a MEK inhibitor led to preferential reduction in the fraction of Bmi1 positive tumor cells and a dramatic decrease in tumor mass [12]. Consistent with these findings cells of the primitive endoderm which will give rise to lung progenitor cells show dependence on mitogenic signaling for self-renewal rather than differentiation [13]. It has previously been shown that the polycomb group protein Bmi1 is a crucial factor for self-renewal in adult stem cells/tumor stem cells through the inhibition of the INK4a/ARF locus encoding p16^Ink4a^/p19^ARF^ ([14], reviewed in [15]). Bmi1 has also recently been identified as a prognostic marker for NSCLC [16], [17] and belongs to a group of factors that have been identified as a death-from-cancer signature in human malignancies [18].

In this study we set out to evaluate Bmi1 function in RAF induced adenoma formation. Because of the restricted life span of Bmi1 gene ablated mice [19] we took advantage of the higher incidence and shorter latency of the BXB11 founder line to examine the Bmi1 dependence of adenoma growth in advanced disease.

Bmi1 ablation via crossing the Bmi1−/− background into the BXB23 and BXB11 founder line revealed that Bmi1 is not required for tumor initiation but for expansion of initiated cells.

## Materials and Methods

### Ethics statement

All animal studies were approved by the Bavarian State authorities for animal experimentation.

### Animals

BXB23 and BXB11 mice were routinely bred in the C57Bl/6 background. For generation of Bmi1−/−BXB23 and Bmi1−/−BXB11 mice SP-C C-RAF BXB mice were crossed with Bmi1+/− mice that had been propagated on a FVB/N background. The resulting Bmi1 heterozygous compound mice were backcrossed with FVB/N/Bmi1+/− mice to generate Bmi1−/− SP-C C-RAF BXB compound mice.

### Reagents and antibodies

Antibodies used for immunohistochemistry/immunofluorescence: Pro SP-C rabbit polyclonal antibody (Chemicon international), CC10 goat polyclonal antibody (Santa Cruz Biotechnology), pan-Cytokeratin rabbit polyclonal antibody (DAKO), Ki-67 mouse monoclonal antibody (Novocastra), C-RAF mouse monoclonal antibody (Santa Cruz Biotechnology), Bmi1 mouse monoclonal antibody (Upstate), p19^ARF^ rabbit polyclonal antibody (Abcam), c-MYC rabbit polyclonal antibody (Santa Cruz Biotechnology), p16^INK4a^ rabbit polyclonal antibody (Santa Cruz Biotechnology), rabbit monoclonal phospho-p44/p42 MAPK Thr 202/Tyr204 (phospho-ERK) antibody (Cell Signaling).

### Microscopy

Fluorescence microscopy was performed using an Openlab software (Improvision, Coventry, UK) controlled DMIRBE microscope (Leica, Wetzlar, Germany) with a 40× Leica oil immersion objective. All images were captured and stored as Openlab LIF files. Images were subsequently processed using power point and Adobe Illustrator software.

### Histopathology and Immunohistochemistry/Immunofluorescence staining

Animals were sacrificed and lungs were fixed under 25 cm water pressure with 4% PBS buffered formalin. Histology was done on formalin-fixed, paraffin-embedded lung specimen. 6 µm-cut sections were deparaffinized, rehydrated in graded alcohols and haematoxylin-eosin (H&E) stained. For quantification of tumor incidence the number of tumors within at least five different areas (310390 µm^2^) per H&E or C-RAF stained lung sections was counted. At least four mice were analyzed for each genotype. Tumor volume was calculated either from H&E stained or anti-C-RAF stained tumors from whole lung sections assuming a spherical tumor. The diameters of the tumors were measured by using a Leica microscope. For quantification of tumor volumes at least four animals per genotype and age were examined: 321 tumors from BXB11, 266 tumors from Bmi1−/−BXB11, 92 tumors from BXB23 and 54 tumors from Bmi1−/−BXB23 animals at the age of two weeks were analyzed. 133 tumors from Bmi1−/−BXB11, 121 tumors from BXB23 and 4 tumors from Bmi1−/−BXB23 animals at the age of three months were analyzed. For the quantification of proliferation Ki67/pro SP-C positive cells in lung sections from mice of the indicated genotype and age were analyzed. A total of at least 1200 pro SP-C positive cells were counted from five randomly selected tumors and analyzed for Ki67 co-staining. For TUNEL staining the Dead End Fluorometric TUNEL-System (Promega) was used. A total of 20 randomly selected tumors from four mice (Bmi1−/−BXB11) and 25 randomly selected tumors from five mice (BXB11) were analyzed. 325 (+/−100) DAPI positive cells per tumor were analyzed for TUNEL positivity. For quantification of cells with phospho-ERK nuclear staining a minimum of 14 tumors from two mice of the indicated genotype and age were analyzed. Semi automated morphological analysis of lung sections from WT and Bmi1−/− animals was carried out as described in [20]. For quantification of pro SP-C/CC10 double positive cells five randomly selected tumors per lung section from a total of 5 animals at the ages of 2 weeks to 3 months were analyzed. Settings for image capture were chosen that allowed the detection of double positive cells at bronchio-alveolar-duct junctions (BADJs).

Immunofluorescence stainings were carried out according to the following basic protocol: Paraffin embedded sections were deparaffinized and rehydrated. For microwave antigen retrieval sections were incubated in 10 mM citrate buffer for 6–23 min. Slides were blocked in appropriate buffer containing either 4% goat or donkey serum, or 1% BSA (exclusively for Bmi1 staining). Incubation with primary antibodies was carried out overnight at 4°C followed by subsequent steps as follows: For pro SP-C/Ki67 co-staining sections were incubated with donkey anti-mouse biotinylated antibody (1∶200) and donkey anti rabbit Cy5 antibody (1∶200) for 90 min at room temperature (RT) sections were subsequently incubated with streptavidin conjugated Alexa 555 (1∶200). For Bmi1 staining sections were incubated with a biotinylated goat anti mouse antibody in blocking buffer for 90 min at RT. After three washes with PBS the sections were incubated with ABC reagent (Vectastain Elite ABX Kit, Vector Labs) for 30 min at RT. Subsequently sections were incubated with Cy5 labeled anti HRP (Horseraddish peroxidase) antibody (1∶100) in blocking buffer. For p19^ARF^ staining sections were incubated with biotinylated donkey anti rabbit antibody (1∶200) 90 min at RT. After three washes with PBS sections were incubated with streptavidin conjugated Alexa 555 (1∶200) in PBS, 0,2% Triton X-100, 90 min at RT. For pro SP-C/CC10 co-staining sections were incubated with donkey anti-goat Alexa 555 (1∶200) and donkey anti rabbit Cy5 (1∶200) antibodies in blocking buffer for 90 min. All stainings were finally counterstained with DAPI before mounting.

For immunohistochemical stainings sections were deparaffinized and rehydrated. For antigen retrieval sections were incubated in 10 mM citrate buffer at 90°C for 10–20 min. Endogenous peroxidase activity was quenched with methanol or PBS containing 3% H_2_O_2_. For c-MYC staining, sections were blocked 60 min with PBS containing 0.2% triton X-100 and 4% goat serum. After primary antibody incubation over night at 4°C sections were incubated with Goat anti rabbit biotinylated secondary antibody at a 1∶200 dilution for 60 min at RT. ABC reagent was applied (Vectastain Elite ABX Kit, Vector Labs) and developed in diaminobenzidine (DAB). Sections were then counterstained with haematoxylin and mounted after dehydration. Phospho p44/p42 MAPK staining was essentially carried out as described for c-MYC with the following changes: TBS, 1% Tween 20 (TBST) was used for washes. Sections were blocked with TBST containing 0.2% Triton X-100 and 5% goat serum. Secondary antibody incubation was carried out using biotinylated goat anti-rabbit antibody in blocking buffer for 60 min. Pan-Cytokeratin stainings were carried out as previously described [8].

### Western blot analysis of whole lung extracts

For preparation of extracts freshly prepared lungs were transferred to a tube containing 500 µl ice cold RIPA buffer (50 mM Tris-HCl, pH 8.0, 150 mM NaCl, 0.1%SDS, 0.5% deoxycholate(DOC), 1% Nonidet P40 with protease inhibitor cocktail). Lungs were homogenized for 5 min using an ultraturax homogenizer in cold environment. Tubes containing the lung homogenate were spun in a cooled centrifuge at 18000 g for 30 min. After centrifugation supernatants were transferred to a fresh tube and aliquots taken for protein determination. The remaining extracts were mixed with 2× Laemmli loading buffer heated to 95°C for 5 min and either stored at −20°C or resolved by SDS-PAGE and transferred to nitrocellulose membranes. After a one hour blocking period in PBS (0.05% Tween, 5% powdered skim milk) blots were incubated overnight with primary antibody, washed three times with PBS and subsequently incubated with a peroxidase coupled secondary antibody. After three times wash in PBS blots were developed.

### RTPCR

RNA was prepared from whole lungs of two weeks old mice using the peqGOLD Trifast RNA preparation kit (peqlab Biotechnologie GmbH) according to the manufacturer instructions. Samples were subjected to DNAse I treatment before preparation of cDNAs using random hexamer primers provided by the First Strand cDNA Synthesis Kit (Fermentas).

Real-time PCR was performed monitoring SYBR green incorporation. As an internal control the RNA levels of the HPRT gene were monitored. Real-time PCR analysis was performed in a Roto-Gene 2000 detection system (Corbet Research). Primer sequences: HPRT: CACAGGACTAGAACACCT; GCTGGTGAAAAGGACCTCT; p19^ARF^: GCGCTCTGGCTTTCGTGAAC; CGTGTCCAGGAAGCCTTCCC; p16^INK4a^: CGTGAGGGCACTGCTGGAAG; ACCAGCGTGTCCAGGAAGCC; p15^INK4b^: CCAGGAAGCCTTCCCGAGCT; GGGGGCAAGTGGAGACGGTG; Cbx7: CGGCCCATTGGTCAGGTCTG; GACCCTCGCCTTGTCATGGC.

## Results

### Development of NSCLC in BXB23 and BXB11 mice

**Figure 1:**
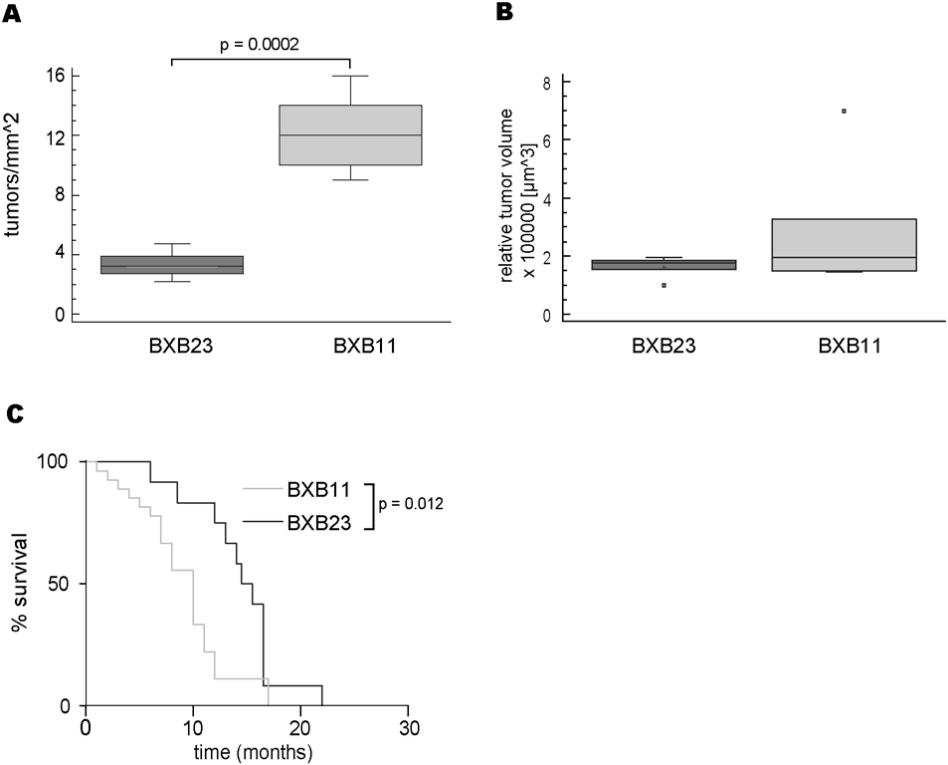
BXB11 mice differ from BXB23 mice with respect to tumor incidence and survival. A) Quantification of tumor incidence in BXB23 and BXB11 lungs at the age of 3 weeks (n = 5 animals, for details see experimental materials). B) Quantification of tumor volume between BXB23 and BXB11. The difference between the two founders was not significant. Data presented as Box-and-Whisker plots. Boxes delineate first and third quartile, Whiskers represent minima and maxima respectively, medians are indicated by solid line within boxes, small squares represent experimental outliers. p-values were calculated using Student's t-test, only p-values indicating significance (p<0,05) are shown. C) Kaplan-Meier plot for survival of BXB11 and BXB23 mice. P-value was calculated using Logrank test.

Lung adenoma formation was compared between BXB23 and BXB11 RAF transgenic founder lines. As judged from H&E staining there was no difference in histopathology of tumors from both lines. However, the number of tumor foci per area was strikingly different. Quantification of tumors showed a 4–5 fold higher incidence in lungs of BXB11 as compared to BXB23 animals (Figure 1A). The basis of this difference between the strains may result from the higher fraction of cells showing nuclear phospho-ERK in BXB 11 mice (Figure S1A and B). Based on the number of tumors per section we calculated the total amount of tumors per lung in the range of 3000 in an average BXB23 lung and 12000–15000 tumors in an average BXB11 lung. On the background of approximately 1–2×10^6^ AT2 cells within the mouse lung [21] we conclude that in both founder lines only a very limited number of AT2 cells serve as tumor initiating cells. This finding is consistent with tumor initiation in a progenitor compartment and resistance of terminally differentiated AT2 cells to transformation although stochastic transgene shutdown cannot be excluded. Comparison of tumor volume between founders did not reveal significant differences (Figure 1B). Tumors in BXB11 are more frequent and therefore more rapidly fill the lung leading to suffocation as evident from the survival curves (Figure 1C).

### Bmi1 promotes tumor growth but not tumor initiation in both the BXB11 and BXB23 mice

**Figure 2:**
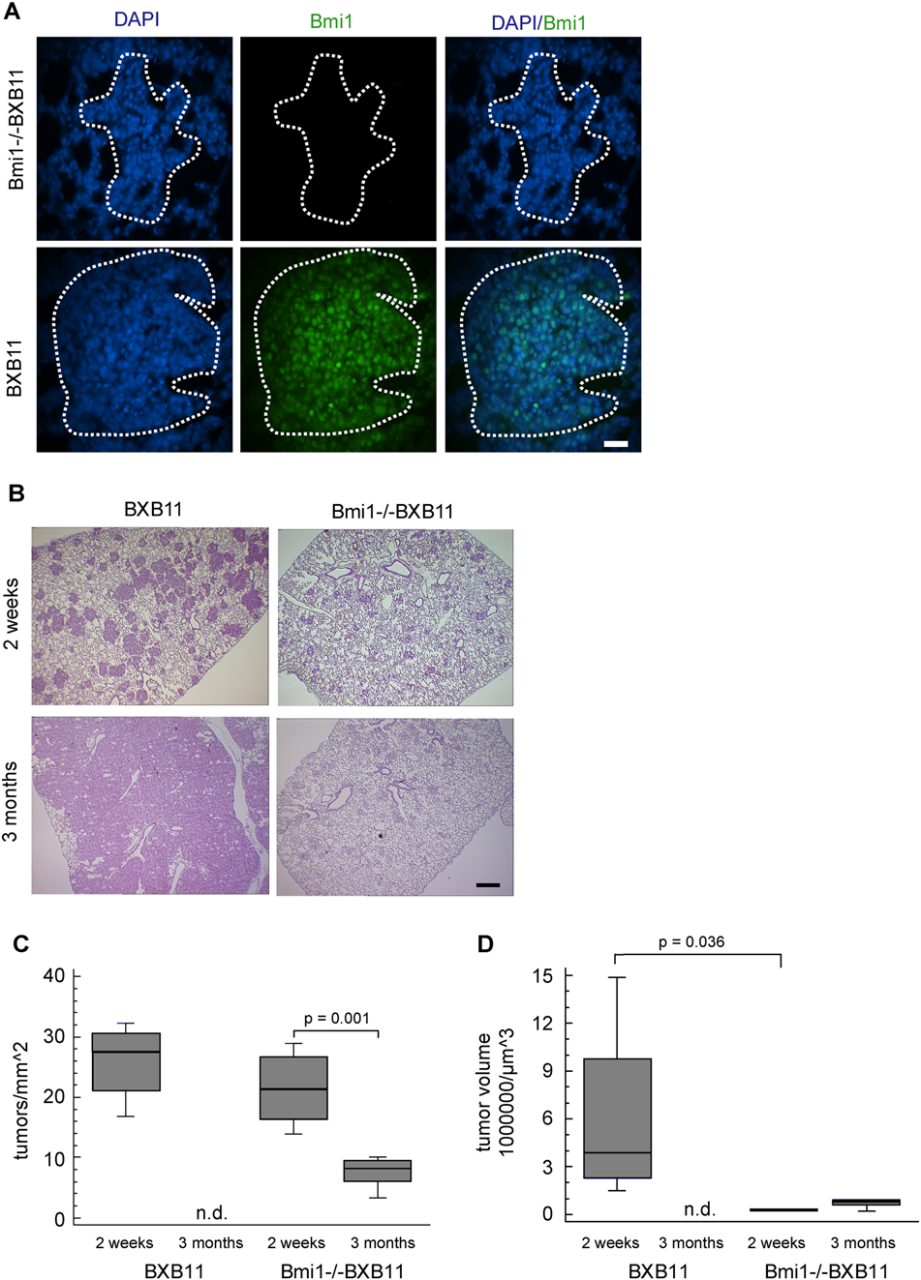
Lack of Bmi1 expression correlates with reduced tumor growth but not incidence in BXB11 mice. A) Paraffin embedded lung sections from BXB11 and Bmi1−/−BXB11 lungs were stained for Bmi1 (green) and DAPI (blue). Dotted white lines delineate tumors. Scale bar = 30 µm. B) H&E staining of lung sections from BXB11 and Bmi1−/−BXB11 mice at the age of two weeks and three months demonstrates reduced tumor burdon in Bmi1−/−BXB11 lungs. Scale bar = 200 µm. C and D) Analysis of tumor incidence and growth within the lungs of BXB11 and Bmi1−/−BXB11 mice. Data presented as Box-and-Whiskers plots, for details of data presentation see figure legend of Figure 1 (n≥4 animals per genotype and age, for details see experimental materials). Note that tumor incidence is increased by a factor of two when compared to Figure 1A) because of the FVB/N genetic background introduced through mating with the Bmi1−/− mice. n.d. = not determined because tumors were confluent. p-values were calculated using Student's t-test only p-values indicating significance are shown.

Based on the observation that Bmi1 is expressed in BXB11 induced tumors and the previously described role of Bmi1 in tumor stem cell self-renewal [14], [22], [23] we decided to further study the role of Bmi1 for the development of C-RAF BXB adenomas (Figure 2A). We generated compound mice expressing the SP-C C-RAF BXB transgene on the Bmi1 knock out background. As the genomic deletion of Bmi1 had no effect on lung development and structure of the adult lung (Figure S2) any alterations in tumor growth can be attributed to Bmi1. The analysis of the Bmi1−/− SP-C C-RAF BXB mice is hampered by the short life span of these mice that is caused by the deletion of Bmi1 [24] and rarely allowed monitoring of tumor development for more than three months.

We therefore first analyzed the effect of Bmi1 ablation on lung tumor initiation in the BXB11 background as the life time of these mice matched that of Bmi1 knock out mice. In contrast to BXB23, BXB11 lungs showed extremely large areas of the lung occupied by tumor tissue at three months of age. Tumor growth was strongly reduced within lungs of Bmi1−/−BXB11 mice (Figure 2B) in two ways, first there was an approximately two fold decrease of tumor numbers between two weeks and three months of age (Figure 2C) and second the volume of surviving tumors decreased (Figure 2D). Similar data was obtained for Bmi1−/−BXB23 compound mice (Figure S3). Though reduced in size the tumors in Bmi1−/−BXB11 lungs showed features of SP-C C-RAFBXB induced adenomas as judged by analysis of cell morphology and staining with two tumor markers (pan-cytokeratin and pro SP-C; Figure S4). Also the shut down of transgene expression as a consequence of Bmi1 ablation can be ruled out as a reason for reduced tumor growth since Bmi1−/−BXB11 tumors stained positive with a C-RAF antibody (Figure S4). Consistently the level of nuclear phospho-ERK was maintained in BXB11 and Bmi1−/−BXB11 from two weeks to three months of age demonstrating that cessation of growth is not caused by a reduction in signaling through the mitogenic cascade (Figure S5).

In summary the ablation of Bmi1 in BXB11 mice leads to strongly reduced tumor growth. The initial number of tumors in BXB11 versus Bmi1−/−BXB11 however, does not show a significant reduction in Bmi1−/−BXB11 lungs, indicating that the specification of tumor initiating cells does not depend on Bmi1.

### Increased cell death and reduced fraction of cells in cycle in Bmi1−/−BXB11 animals

**Figure 3:**
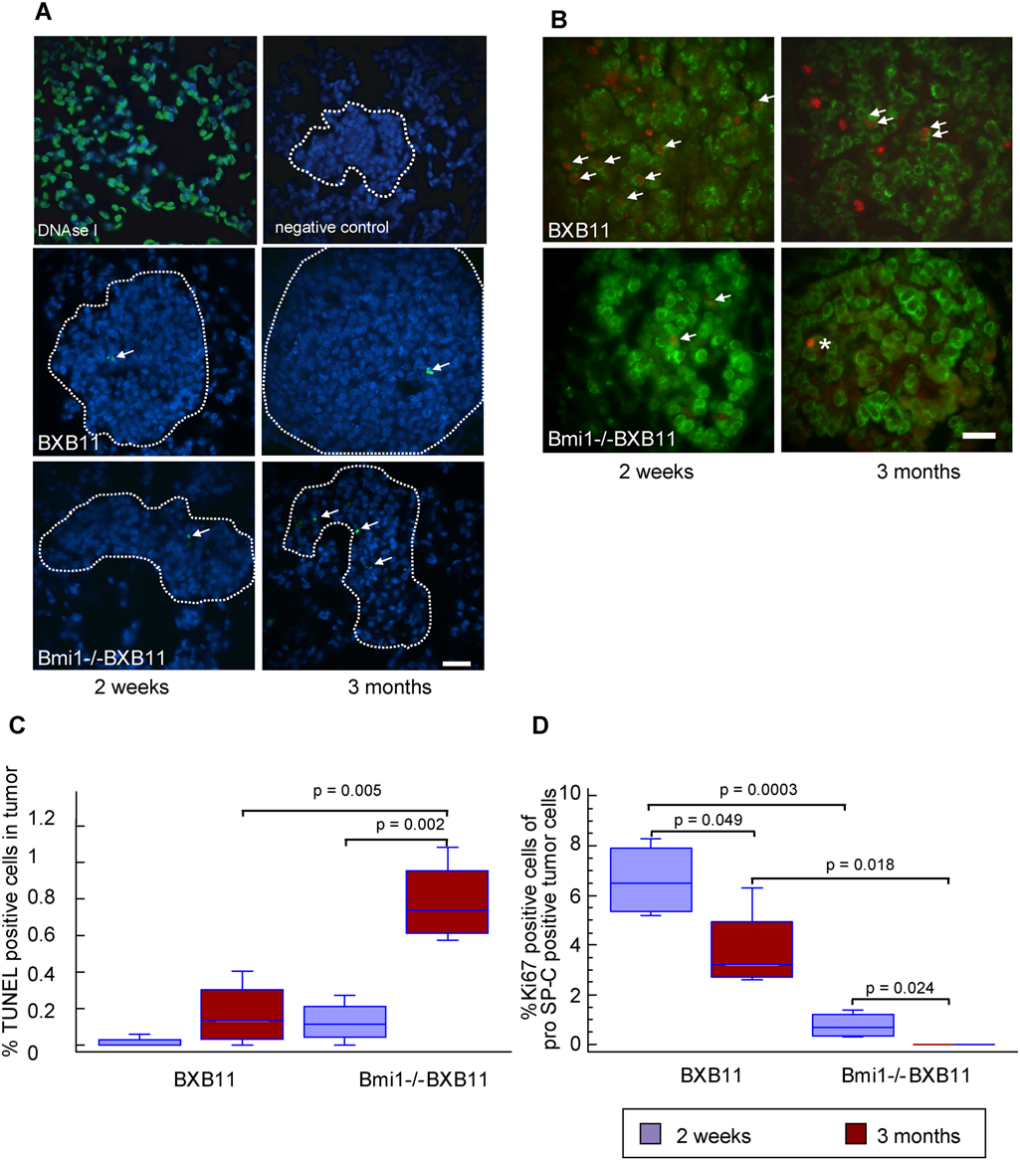
Increased apoptosis and reduced proliferation in Bmi1−/−BXB11 tumors. A) TUNEL staining of paraffin embedded lung sections from Bmi1−/−BXB11 and BXB11 mice. DNase I treated lung sections were used as positive control. Sections treated without terminal deoxynucleotidyl transferase served as negative control. Dotted white lines delineate tumors. Genotypes and ages as indicated. Arrows indicate apoptotic (green) cells. Scale bar = 30 µm. B) Ki67 (red)/pro SP-C (green) co- immunofluorescence staining of lung sections from BXB11 and Bmi1−/−BXB11 mice. Arrows indicate cycling tumor cells. “*” indicates staining artifact. Scale bar = 30 µm. C) Quantification of TUNEL positive cells in lung tumors of two weeks and three months old BXB11 and Bmi1−/−BXB11 mice (n = 4 animals per genotype), for details see materials and methods. Data presented as Box-and-Whiskers plot for details of data presentation see figure legend of Figure 1. D) Quantification of Ki-67/pro SP-C double positive cells. At least five tumors from five different animals were analyzed. p-values were calculated using Student's t-test, only p-values indicating significance are shown.

The strongly reduced tumor growth and the loss of tumors over time in both founder lines with abrogated Bmi1 expression is either a consequence of a block in cell cycle progression or increased cell death or a combination of both. Bmi1 has previously been implicated in promoting tumor cell survival and cell cycle progression in a tissue culture model system [25], [26]. TUNEL staining showed a significant increase in the apoptotic cell ratio in adenomas of three months old Bmi1−/−BXB11 mice (Figure 3A and 3C). This indicates that apoptosis is a late event in Bmi1−/−BXB11 adenomas. Next we examined growth rate. We performed Ki-67/pro SP-C co-staining on lungs prepared from two weeks and three months old Bmi1−/−BXB11 and BXB11 animals. The fraction of cells in cycle is strongly reduced upon Bmi1 ablation. Moreover as evident from the BXB11 control at the age of three months the rate of growth of these tumors has also decreased suggesting either a limited replicative lifespan for BXB11 transformed AT2 cells or other levels of growth control (Figure 3B and 3D).

In conclusion both tumor cell survival and cell proliferation are affected in BXB11 mice with Bmi1 deletion and can account for the observed reduced tumor growth.

### Bmi1 dependent expression of p16 ^INK4a^ and p19^ARF^

**Figure 4:**
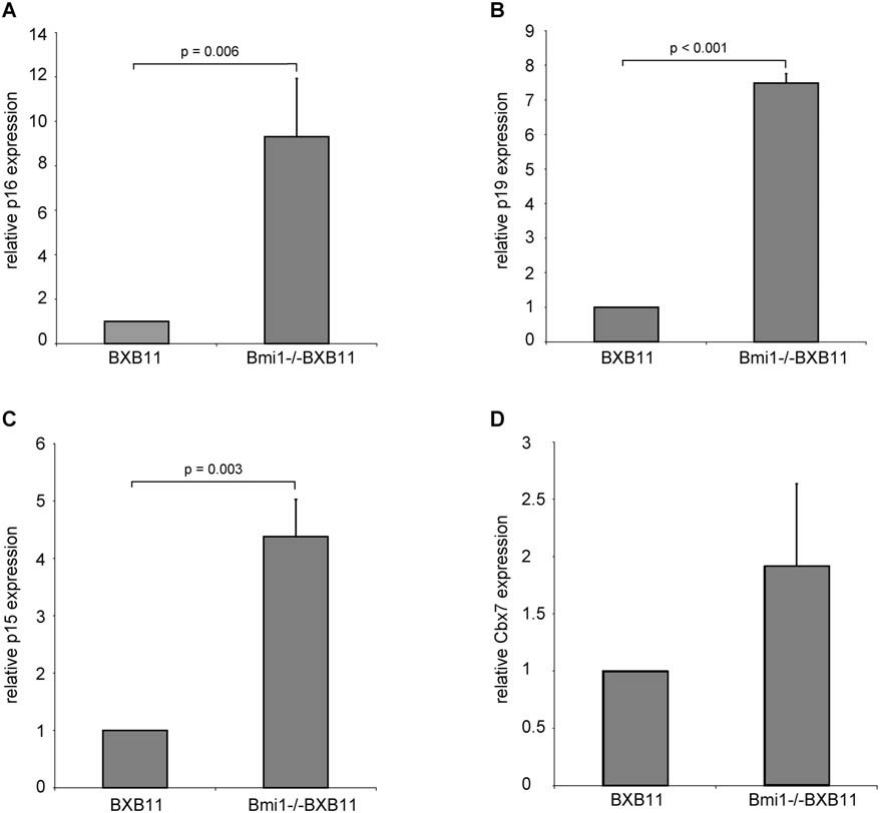
p15^INK4b^, p16^INK4a^, p19^ARF^ and Cbx7 expression in lungs of BXB11 and Bmi1−/−BXB11 mice. A–D) Semi-quantitative real time PCR analysis of total RNA from whole lungs of two weeks old mice, genotypes as indicated. BXB11 levels were set to one. Data are representative of two independent sets of analysis. Data show standard deviations from triplicate values. p-values were calculated using Student's t-test, only p-values indicating significance are shown. A) Quantification of p16^INK4a^ expression levels B) Quantification of p19^ARF^ RNA expression levels. C) Quantification of p15^INK4b^ expression levels. D) Quantification of Cbx7 expression levels.

Bmi1 is implicated in cdki regulation. Most prominently p16^INK4a^, p19^ARF^ have been shown to be negatively regulated by Bmi1 [27]. Therefore altered p16^INK4a^/p19^ARF^ expression levels could account for the reduced number of surviving and cycling cells in Bmi1−/−BXB11 tumors. We first analyzed expression of mRNA levels of p16^INK4a^ by semi-quantitative RTPCR in lungs of two weeks old BXB11 and Bmi1−/−BXB11 mice (Figure 4A). Expression levels of p16^INK4a^ were markedly increased in Bmi1−/−BXB11 lungs. Western blot analysis of whole lung lysates confirmed this result on the protein level (Figure S6A).

Next we analyzed the mRNA expression levels of p19^ARF^. As in the case of p16^INK4a^, p19^ARF^ and p15^INK4b^ expression levels were up-regulated in lungs of Bmi1−/−BXB11 when compared to BXB11 (Figure 4B and 4C). In contrast to p16^INK4a^ that was not altered between wild type and BXB11 mice, we found elevated levels of p19^ARF^ RNA expression also in BXB11 lungs (data not shown). Another epigenetic regulator of the INK4a locus, Cbx7 was not significantly altered in expression upon Bmi1 ablation in BXB11 mice (Figure 4D). Immunofluorescence staining of Bmi1−/−BXB11 and BXB11 lungs showed that p19^ARF^ is expressed in lung tumors of animals of both genotypes. Differences in the subnuclear distribution of p19^ARF^ are, however, evident when Bmi1−/−BXB11 and BXB11 tumors are compared. While the ablation of Bmi1 is associated with a multi focal nuclear staining pattern of p19^ARF^ in almost all tumor cells it is mostly confined to a single locus within the nuclei of tumors in the presence of Bmi1 (Figure S7).

In conclusion we find reduced proliferation in late stage p19^ARF^ positive BXB11 tumors that might be interpreted as part of a senescence phenotype. A potential explanation for this mild effect may be c-MYC expression that we find in BXB11 tumor cells (Figure S6B) and that has been recently described to revert a sensescence phenotype in B-RAF transformed melanomas [28]. In the absence of Bmi1 however, expression of INK4a/Arf was more pronounced even though c-MYC expression was maintained (Figure S6B) eventually leading to cessation of proliferation. Thus under these conditions a c-MYC rescue may no longer operate.

### No evidence for bronchio-alveolar-stem cells (BASCs) in lung tumors independent of Bmi1

**Figure 5:**
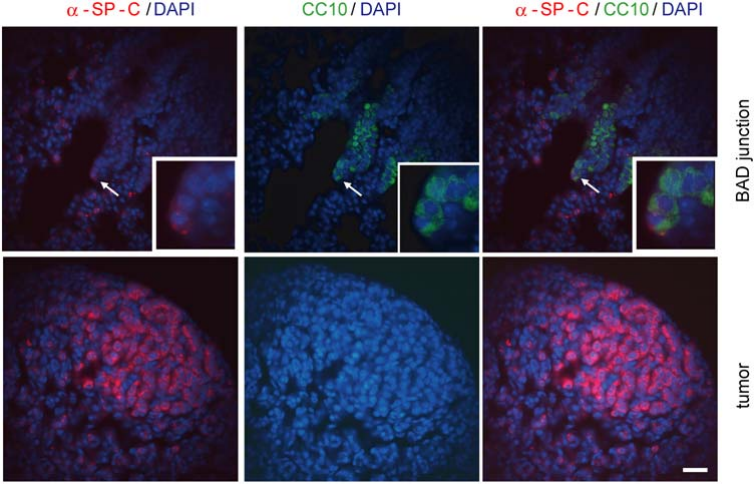
No evidence for BASCs in BXB lung tumors. Paraffin embedded lung sections from BXB11 mice were stained for pro SP-C (red) and for CC10 (green) to detect double positive bronchio alveolar stem cells (BASCs). A representative BASC cell that is located at a bronchio alveolar duct junction (BADJ) is indicated by white arrow. Scale bar = 30 µm.

We next addressed the contribution of BASC cells to SP-C C-RAF BXB tumor formation. To this end we performed pro SP-C/CC10 double staining and analyzed BXB11 tumors for the presence of double positive cells. For this analysis image acquisition settings were chosen that allowed identification of BASCs at BADJs. As illustrated in Figure 5, no double positive cells could be observed in tumors.

In summary this data indicates that the pool of Bmi1 independent tumor cells that presumably represents tumor initiating cells does not represent BASCs.

## Discussion

In the current study we employed two transgenic founder lines with lung targeted expression of C-RAF BXB for the dependence of adenoma initiation and growth on the stem cell self-renewal factor Bmi1. We show that BXB11 has a four fold higher incidence and shorter latency of adenomas allowing examination of advanced disease in the absence of Bmi1. Depletion of Bmi1 in both the BXB23 and BXB11 background led to a pronounced decrease in tumor growth rate and an increase in cell death within the adenomas. In contrast no significant reduction in tumor initiating events could be observed in both founder lines, arguing for Bmi1 being an important factor for tumor expansion rather then initiation.

Based on the observation, that Bmi1 is expressed in the C-RAF BXB initiated lung adenomas and that Bmi1 is a target of RAF signaling [19] we decided to investigate adenoma formation in the absence of Bmi1. The abrogation of Bmi1 expression in the BXB23 and BXB11 background did not yield a significant reduction in tumor initiation events as defined by the number of tumors that were formed in the various genetic combinations by two weeks of age. Analysis of lung adenoma cells in the absence or presence of Bmi1 did not reveal differences at the morphological and at cell marker expression level. Therefore the absence of Bmi1 did not affect the process of tumor initiation. These results are consistent with a previously published report showing Bmi1 independent glioma initiation albeit in the absence of INK4a [29]. The absence of Bmi1 did not affect the identity of the resulting tumor cells with respect to morphology and expression of a limited set of markers. These results also indicate that Bmi1 is not required for the specification of a tumor initiating cell within the lung. Phospho-ERK levels may compensate Bmi1 loss for short term but not long term self renewal divisions of initiated cells as we observed significant tumor growth in Bmi1−/−BXB11 showing higher nuclear phospho-ERK followed by cessation of cell division at late time points (three months of age) when nuclear phosphor-ERK was still present. In the course of our studies we also did not observe changes in lung morphology in Bmi1−/− mice indicating that Bmi1 is not a crucial factor for proper lung development. We found a strong up-regulation of p19^ARF^, p16^INK4a^ and, p15^INK4b^ in whole lung RNA preparations of Bmi1−/− animals as compared to wild type littermates. The expression of p19^ARF^ can not exclusively be attributed to lung infiltrating cells of the hematopoietic system since we do see pronounced p19^ARF^ expression in AT2 cells and cells lining the bronchi in Bmi1−/− animals (data not shown). Obviously, expression of cdkis does not impair normal lung cell function. This finding is of interest concerning off target effects in possible future therapeutic strategies targeting Bmi1 in lung cancer.

Cdkis encoded at the INK4a locus are implicated in inhibition of cell cycle progression and control of cell survival [30]–[32]. In line with this we do see reduced tumor cell cycle progression in Bmi1−/−BXB11 tumors and an increase in cells undergoing apoptosis albeit with some delay. These findings are in line with previous results published by Liu and colleagues [25] showing *in vitro* a specific effect of Bmi1 on tumor cell survival.

In agreement with previous reports showing p19^ARF^ up-regulation in response to oncogenic hyperproliferative signals [33]–[35], expression of p19^ARF^ was not restricted to the Bmi1−/− background but could also be observed to a lesser extent in BXB11 adenomas. We noted that p19^ARF^ localized to only one to two distinct nuclear foci within BXB11 adenomas. This distribution could not be observed in Bmi1−/−BXB11 adenomas where p19^ARF^ localized throughout the nucleus in many distinct spots. The difference in distribution could be due to different p19^ARF^ expression levels within individual tumor cells. Changes in subnuclear distribution of p19^ARF^ have, however, also been observed as a consequence of DNA damage in cell culture experiments [36]. Whether DNA damage is increased within Bmi1−/−BXB11 tumors and accumulates during the three months observation period thus leading to delayed apoptosis (Figure 3A and C) is currently under investigation.

The increased p19^ARF^ expression is potentially relevant with respect to the observation that the relative number of cycling cells in BXB11 tumors decreased about 50% within 2.5 months. Intrinsic and extrinsic factors could account for this observation. For example the C-RAF BXB adenoma cells could have a limited replicative life span, possibly through negative feed back regulation as shown to be the case for constitutive activation for the mitogenic cascade [37]. Alternatively, growth inhibitory factors might accumulate in late tumor tissues due to crowding.

Induction of NSCLC in mouse lung has also been examined by conditional expression of mutant K-Ras [4]. In this system BASCs have been described to be involved in lung tumor formation. Specifically it has been reported that K-Ras amplifies BASCs at bronchiolar alveolar duct (BAD) junctions and that these transformed stem cells form a stream that supplies lung tumor foci. SP-C promoter controlled expression of RAF clearly does not expand BASCs at BAD junctions yet is very efficient in giving rise to NSCLC. Consistently we did not observe any BASCs within RAF driven tumors that infrequently localize in the vicinity of BAD junctions indicating that the histogenesis of NSCLC in these two tumor models may differ. Nevertheless both models show dependence of tumor expansion on Bmi1 by a mechanism that mainly involves the INK4a/Arf locus [38]. These data suggest that tumors in both mouse models of NSCLC may depend on the same tumor stem cell. If this were to be the case then K-Ras tumors should be sensitive to the mitogenic cascade blocker CI-1040 that we have previously shown to dramatically decrease tumor burden by differential reduction of Bmi1 positive cells in foci [12].

In summary, our results and the results of others highlight the role of Bmi1 as a crucial factor for tumor cell expansion and therefore as an attractive target for tumor intervention.

## Supporting Information

Figure S1 phospho-ERK expression in BXB23 and BXB11 lung tumors. A) Quantification of nuclear phospho-ERK staining shown in B). Data is presented as Box-and-Whiskers plot for details see legend of Figure 1. B) Immunohistochemistry for phospho-ERK. White arrows indicate nuclear phospho-ERK staining (dark brown). Hematoxylin (blue) was used as counter stain. Scale bar = 50 µm.
(7.36 MB TIF)

Figure S2 Bmi1−/− and WT lungs are morphologically indistinguishable. Semi automated analysis of lung sections from four months old WT and Bmi1−/− animals. A) Examples of analysed sections. B) Quantification of average air content (air[%]), septum thickness (wall[Âµm]), mean linear intercept (MLI). n = 2 for each genotype.
(6.02 MB TIF)

Figure S3 Tumor growth but not incidence depends on Bmi1 in BXB23 and Bmi1−/−BXB23 mice. A) Representative H&E stainings of lung sections from four months old BXB23 and Bmi1−/−BXB23 mice. B and C) Analysis of tumor incidence and growth within the lungs of Bmi1−/−BXB23 and BXB23 mice. Data presented as Box-and-Whiskers plots, for details of data presentation see figure legend of Figure 1 (n≥3 animals per genotype and age, for details see experimental materials). p-values were calculated using Student's t-test, only p-values indicating significance are shown. Scale bar = 100 µm.
(4.16 MB TIF)

Figure S4 pan-Cytokeratin SP-C and C-RAF expression in lung adenomas of Bmi1−/−BXB11 and BXB11 mice. Representative immunohistological/immunofluorescence stainings of lung sections from two weeks old animals. Genotypes as indicated. Sections were stained for pan-cytokeratin (brown), pro SP-C (red) and C-RAF (red) as described in the materials and methods section. Pan-cytokeratin staining was counterstained with hematoxylin (blue), pro SP-C and C-RAF stainings were counterstained with DAPI (blue). Scale bar = 50 µm.
(9.77 MB TIF)

Figure S5 phospho-ERK levels do not differ between early and late lung tumors in BXB11 and Bmi1−/−BXB11 mice. A) Quantification of nuclear phospho-ERK staining shown in B). Data is presented as Box-and-Whiskers plot for details see legend of Figure 1. B) Immunohistochemistry for phospho-ERK. White arrows indicate nuclear phospho-ERK staining (dark brown). Hematoxylin (blue) was used as counter stain. Scale bar = 50 µm.
(5.55 MB TIF)

Figure S6 p16^INK4a^ and c-Myc expression in BXB11 and Bmi1−/−BXB11 lungs. A) Top panel: EtBr stained agarose gel showing PCR products of Bmi1 wild type allele genotyping. Genomic DNA was used as template. Lower panels: Western blot analysis of whole lung extracts. Antibodies used for blotting are indicated on the left. Positions of C-RAF and C-RAF BXB are indicated by arrows on the right. In accordance with lower tumor load in Bmi1−/−BXB11 lungs, lower C-RAFBXB protein levels can be detected in these lungs. Significant levels of p16^INK4a^ can only be detected in extracts from Bmi1−/−BXB11 lungs. GAPDH was used as loading control. B) Immunohistological c-Myc staining (brown) of lung sections from three months old animals. Genotypes as indicated. Staining was counterstained with hematoxylin (blue). Scale bar = 100 µm.
(6.99 MB TIF)

Figure S7 Differential subnuclear distribution of p19^ARF^ in BXB11 versus Bmi1−/−BXB11 tumors. Immunofluorescence staining for p19^ARF^ (red). Lung sections of 3 weeks old Bmi1−/−BXB11 and BXB11 mice were used for analysis. Sections were counterstained with DAPI (blue). Arrows point to individual nuclei. Inserts represent segment magnifications of nucleus indicated by arrow head. Scale bar = 25 µm.
(8.48 MB TIF)
