# Supplementary figures and images for: Polycomb Group Protein Bmi1 Is Required for Growth of RAF Driven Non-Small-Cell Lung Cancer
Source: PLoS One. 2009 Jan 19;4(1):e4230. doi: 10.1371/journal.pone.0004230 (PMC2626631; doi:10.1371/journal.pone.0004230)

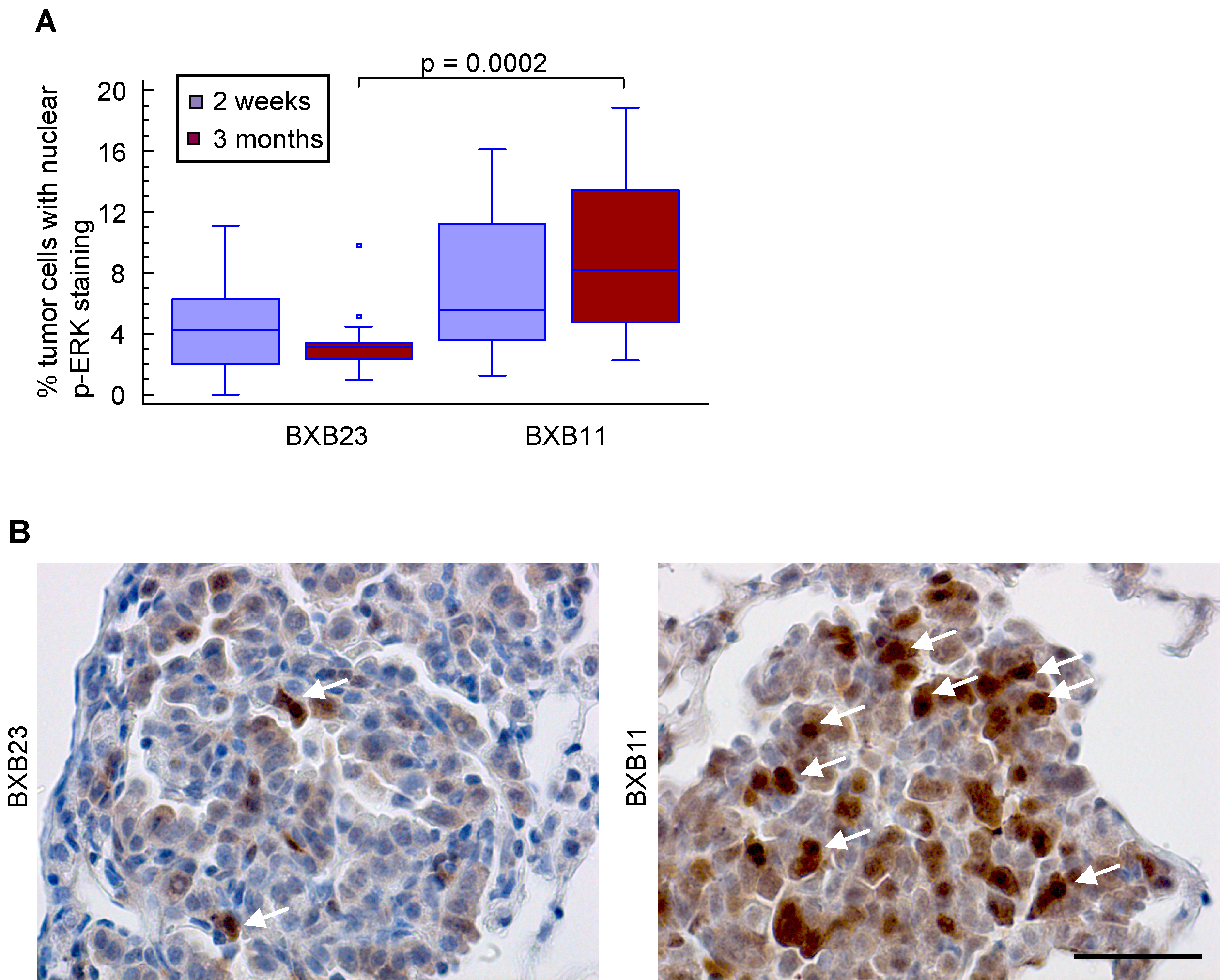

Supplement: Figure S1 — phospho-ERK expression in BXB23 and BXB11 lung tumors. A) Quantification of nuclear phospho-ERK staining shown in B). Data is presented as Box-and-Whiskers plot for details see legend of Figure 1. B) Immunohistochemistry for phospho-ERK. White arrows indicate nuclear phospho-ERK staining (dark brown). Hematoxylin (blue) was used as counter stain. Scale bar = 50 µm. (7.36 MB TIF) [file pone.0004230.s001.tif]

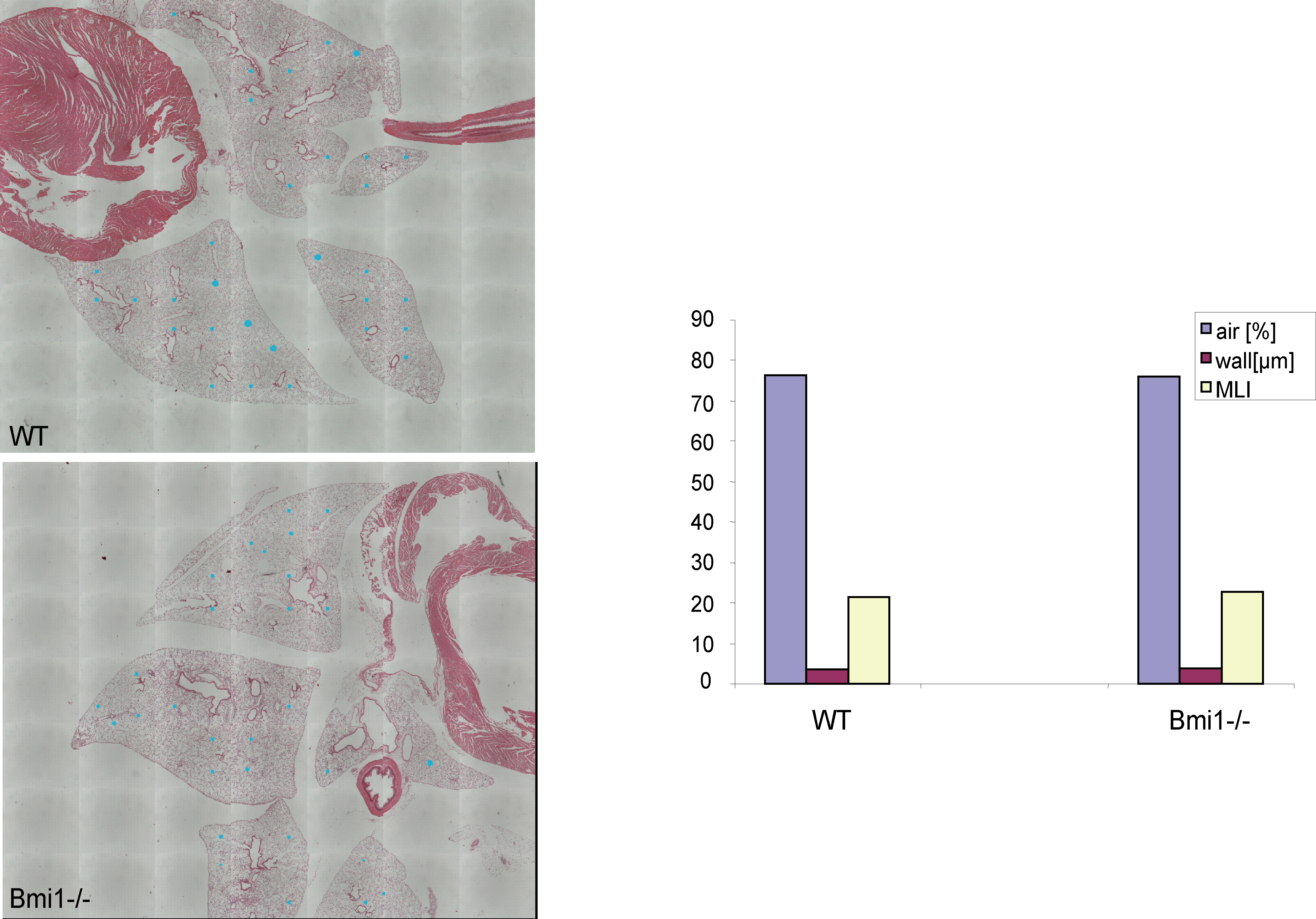

Supplement: Figure S2 — Bmi1−/− and WT lungs are morphologically indistinguishable. Semi automated analysis of lung sections from four months old WT and Bmi1−/− animals. A) Examples of analysed sections. B) Quantification of average air content (air[%]), septum thickness (wall[Âµm]), mean linear intercept (MLI). n = 2 for each genotype. (6.02 MB TIF) [file pone.0004230.s002.tif]

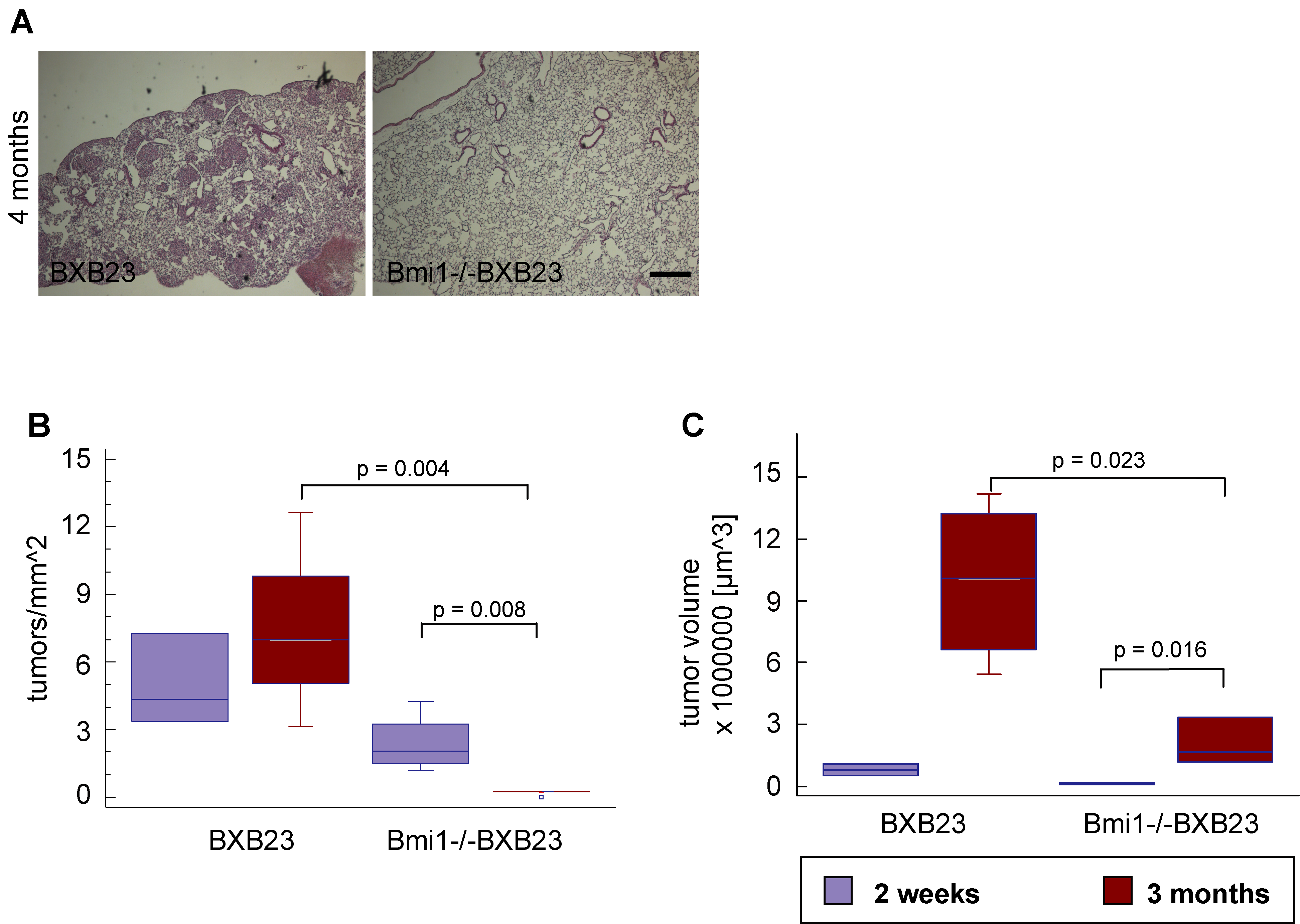

Supplement: Figure S3 — Tumor growth but not incidence depends on Bmi1 in BXB23 and Bmi1−/−BXB23 mice. A) Representative H&E stainings of lung sections from four months old BXB23 and Bmi1−/−BXB23 mice. B and C) Analysis of tumor incidence and growth within the lungs of Bmi1−/−BXB23 and BXB23 mice. Data presented as Box-and-Whiskers plots, for details of data presentation see figure legend of Figure 1 (n≥3 animals per genotype and age, for details see experimental materials). p-values were calculated using Student's t-test, only p-values indicating significance are shown. Scale bar = 100 µm. (4.16 MB TIF) [file pone.0004230.s003.tif]

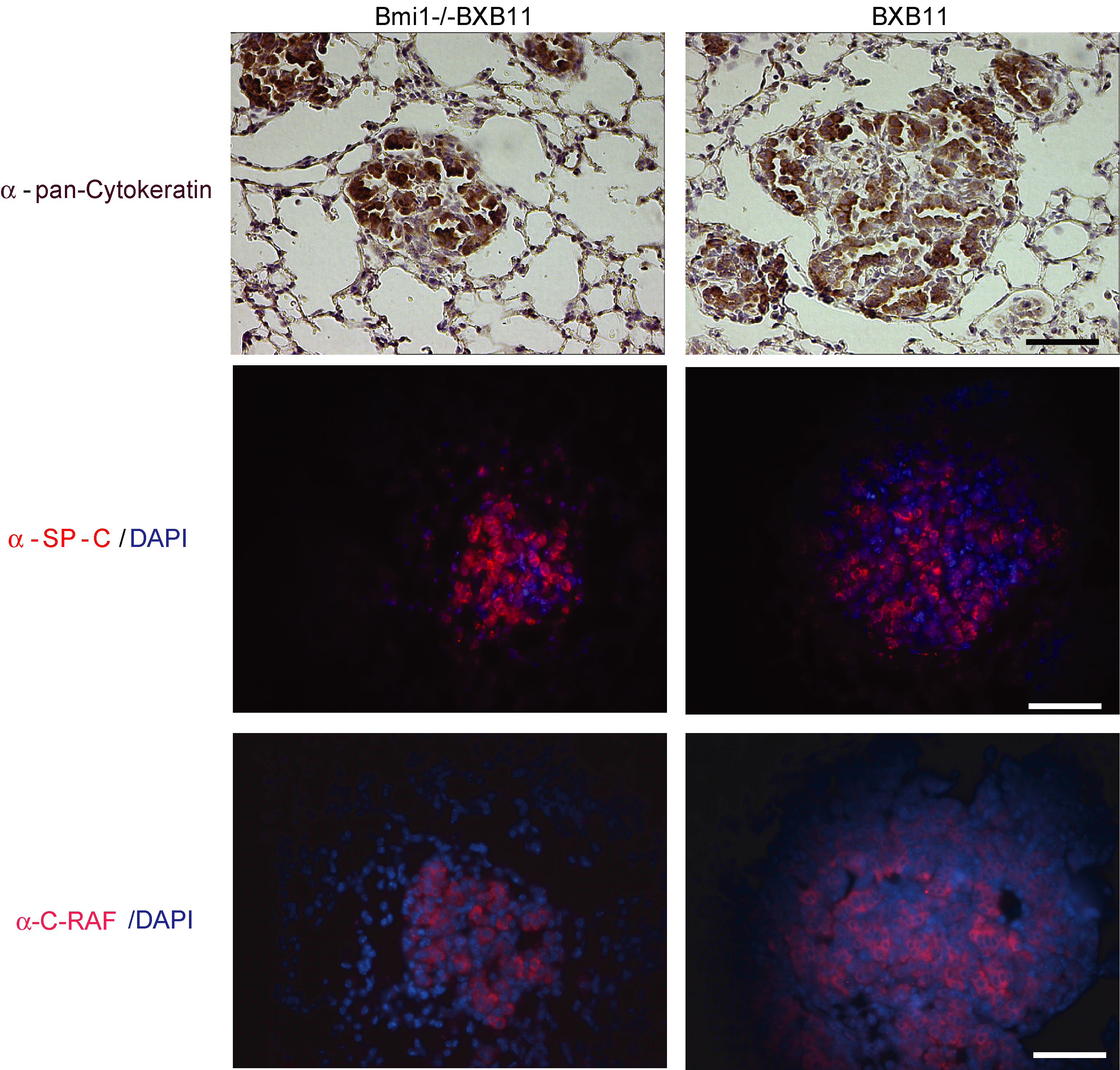

Supplement: Figure S4 — pan-Cytokeratin SP-C and C-RAF expression in lung adenomas of Bmi1−/−BXB11 and BXB11 mice. Representative immunohistological/immunofluorescence stainings of lung sections from two weeks old animals. Genotypes as indicated. Sections were stained for pan-cytokeratin (brown), pro SP-C (red) and C-RAF (red) as described in the materials and methods section. Pan-cytokeratin staining was counterstained with hematoxylin (blue), pro SP-C and C-RAF stainings were counterstained with DAPI (blue). Scale bar = 50 µm. (9.77 MB TIF) [file pone.0004230.s004.tif]

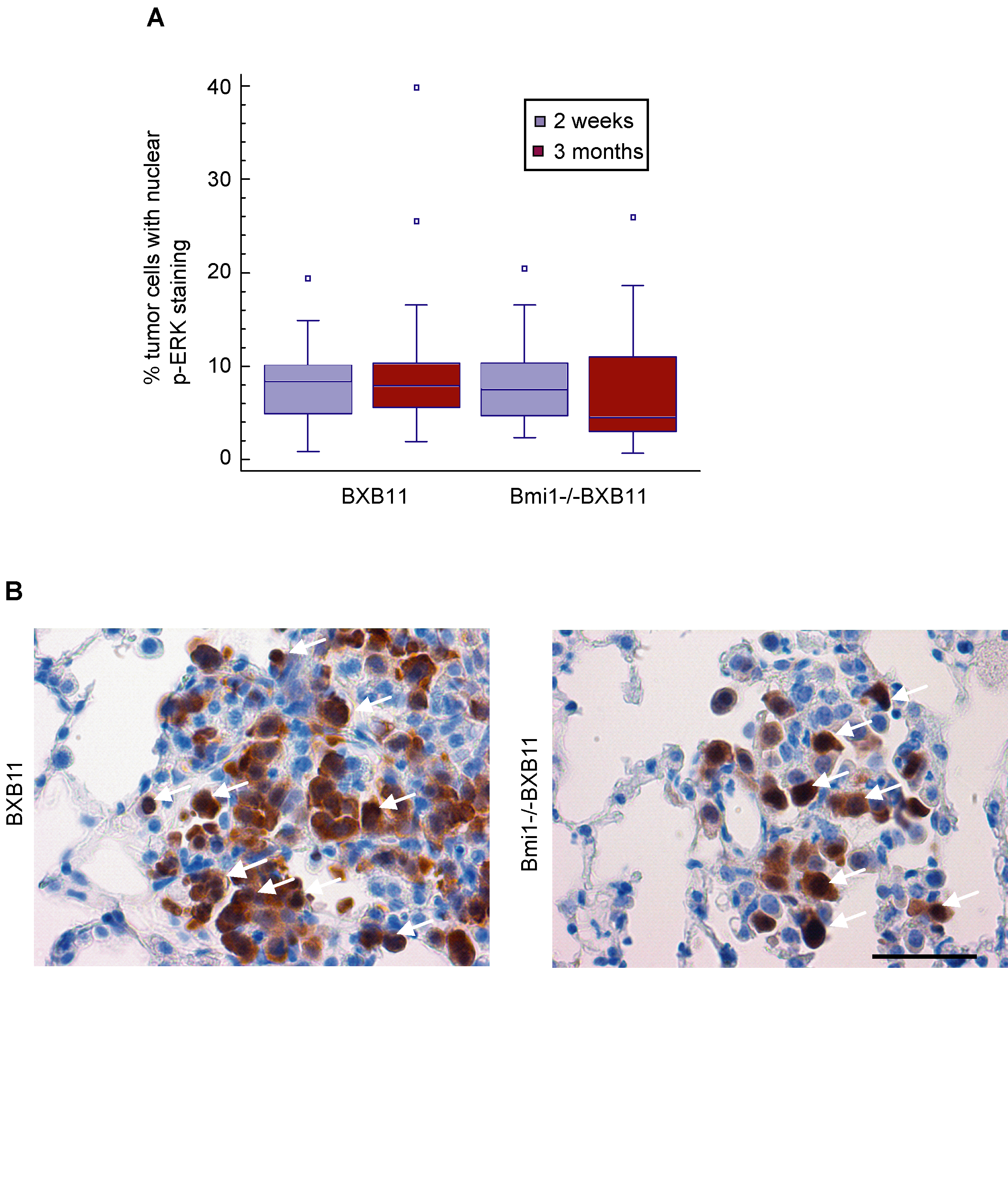

Supplement: Figure S5 — phospho-ERK levels do not differ between early and late lung tumors in BXB11 and Bmi1−/−BXB11 mice. A) Quantification of nuclear phospho-ERK staining shown in B). Data is presented as Box-and-Whiskers plot for details see legend of Figure 1. B) Immunohistochemistry for phospho-ERK. White arrows indicate nuclear phospho-ERK staining (dark brown). Hematoxylin (blue) was used as counter stain. Scale bar = 50 µm. (5.55 MB TIF) [file pone.0004230.s005.tif]

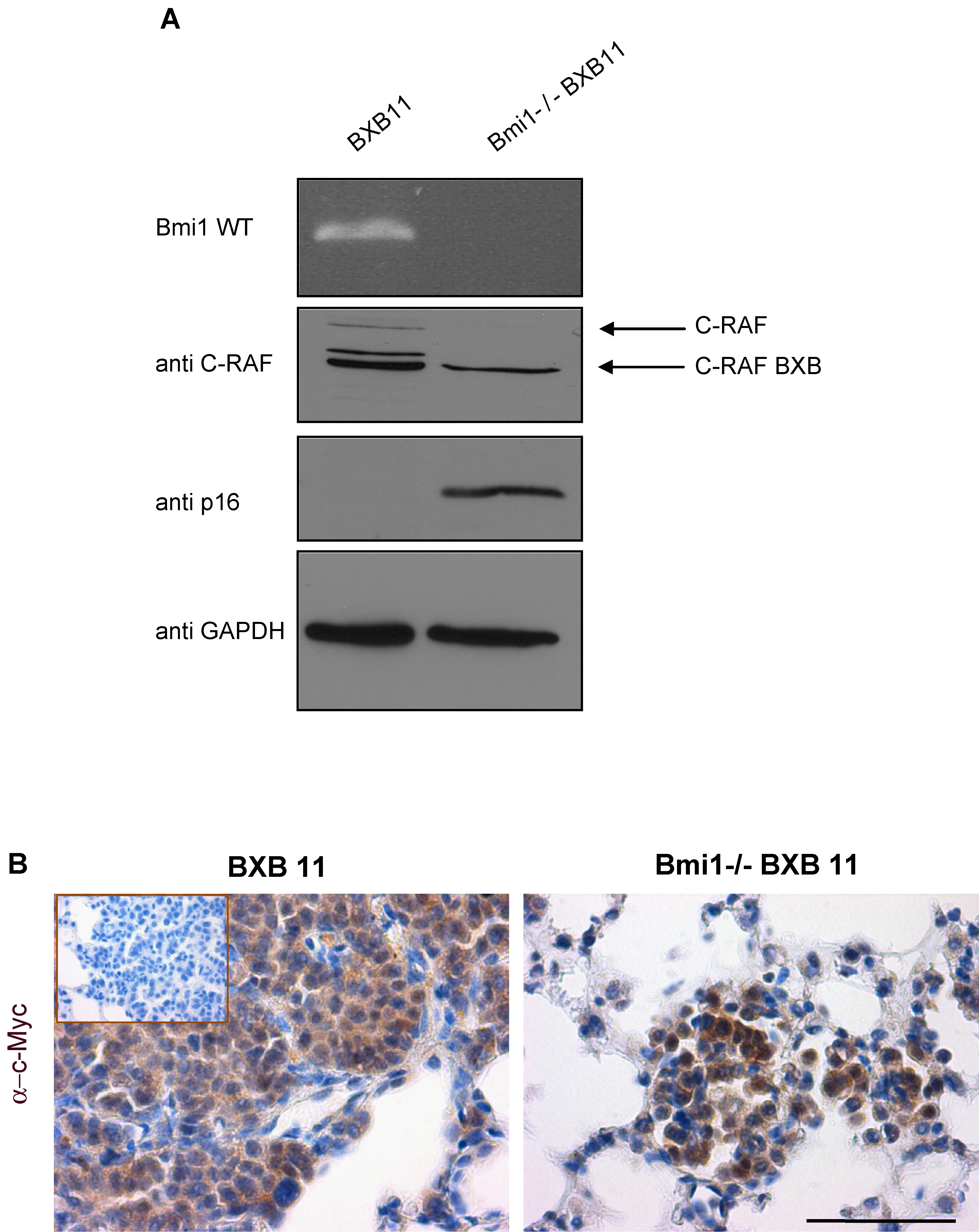

Supplement: Figure S6 — p16INK4a and c-Myc expression in BXB11 and Bmi1−/−BXB11 lungs. A) Top panel: EtBr stained agarose gel showing PCR products of Bmi1 wild type allele genotyping. Genomic DNA was used as template. Lower panels: Western blot analysis of whole lung extracts. Antibodies used for blotting are indicated on the left. Positions of C-RAF and C-RAF BXB are indicated by arrows on the right. In accordance with lower tumor load in Bmi1−/−BXB11 lungs, lower C-RAFBXB protein levels can be detected in these lungs. Significant levels of p16INK4a can only be detected in extracts from Bmi1−/−BXB11 lungs. GAPDH was used as loading control. B) Immunohistological c-Myc staining (brown) of lung sections from three months old animals. Genotypes as indicated. Staining was counterstained with hematoxylin (blue). Scale bar = 100 µm. (6.99 MB TIF) [file pone.0004230.s006.tif]

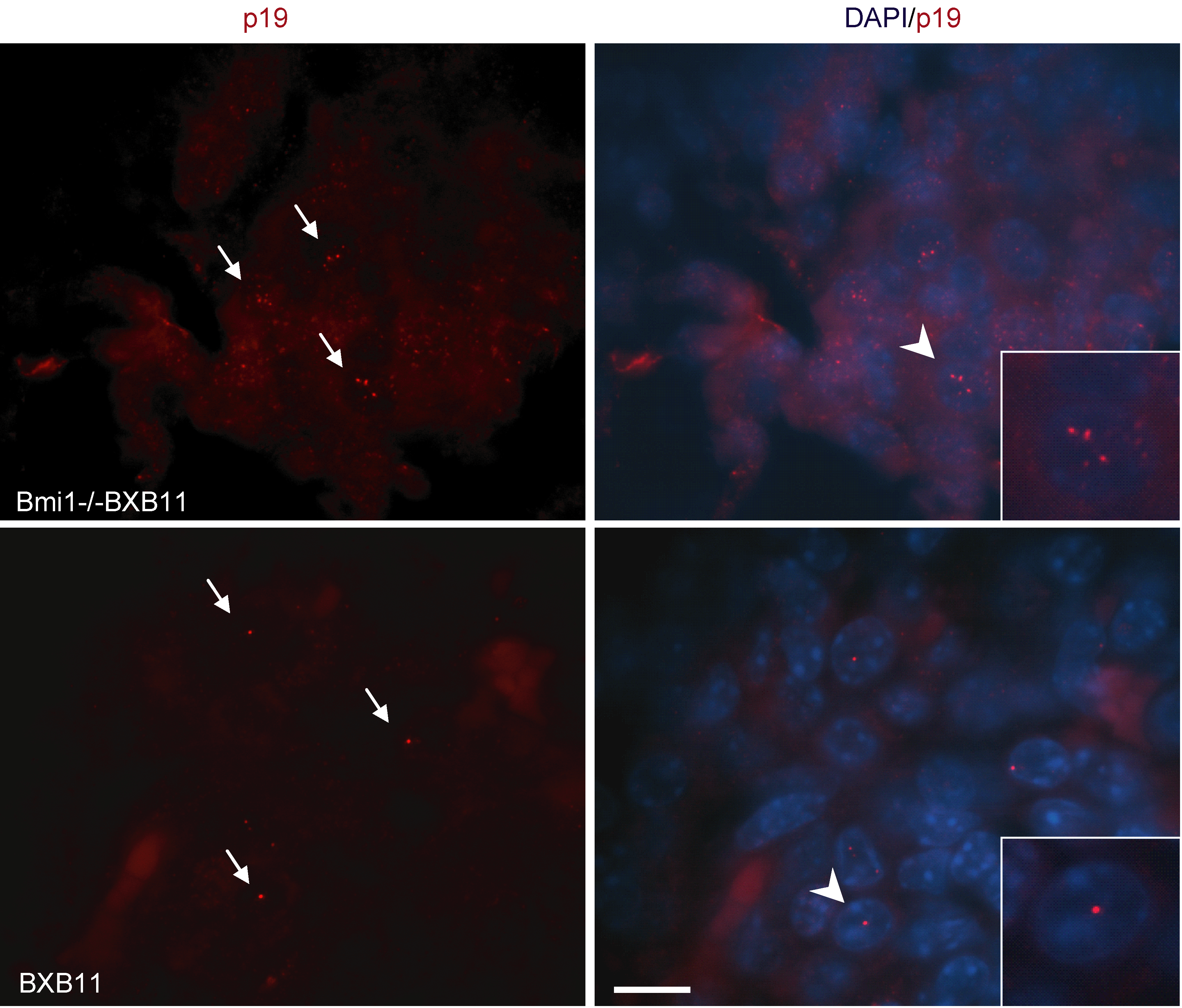

Supplement: Figure S7 — Differential subnuclear distribution of p19ARF in BXB11 versus Bmi1−/−BXB11 tumors. Immunofluorescence staining for p19ARF (red). Lung sections of 3 weeks old Bmi1−/−BXB11 and BXB11 mice were used for analysis. Sections were counterstained with DAPI (blue). Arrows point to individual nuclei. Inserts represent segment magnifications of nucleus indicated by arrow head. Scale bar = 25 µm. (8.48 MB TIF) [file pone.0004230.s007.tif]
